# Imaging of changes in copper trafficking and redistribution in a mouse model of Niemann-Pick C disease using positron emission tomography

**DOI:** 10.1007/s10534-019-00185-5

**Published:** 2019-03-07

**Authors:** Julia Baguña Torres, Zilin Yu, Jayanta Bordoloi, Kavitha Sunassee, David Smith, Claire Smith, Oscar Chen, Rupert Purchase, Karin Tuschl, John Spencer, Frances Platt, Philip J. Blower

**Affiliations:** 1 School of Biomedical Engineering and Imaging Sciences, King’s College London, St Thomas’ Hospital, London, SE1 7EH UK; 2 Department of Pharmacology, University of Oxford, Mansfield Road, Oxford, OX1 3QT UK; 3 Department of Chemistry, School of Life Sciences, University of Sussex, Falmer, Brighton, BN1 9QJ UK; 4 MRC Centre for Developmental Neurobiology IoPPN, King’s College London, London, SE1 1UL UK; 5 Department of Cell and Developmental Biology, University College London, London, WC1E 6BT UK

**Keywords:** Niemann-Pick C, Copper-64, Positron emission tomography, Autoradiography, Copper dyshomeostasis

## Abstract

Niemann-Pick C disease (NPC) is an autosomal recessive lysosomal storage disorder resulting from mutations in the *NPC1* (95% of cases) or *NPC2* genes. Disturbance of copper homeostasis has been reported in NPC1 disease. In this study we have used whole-body positron emission tomography (PET) and brain electronic autoradiography with copper-64 (^64^Cu), in the form of the copper(II) bis(thiosemicarbazonato) complex ^64^Cu-GTSM, to image short-term changes in copper trafficking after intravenous injection in a transgenic mouse model of NPC1 disease. ^64^Cu-GTSM is taken up in all tissues and dissociates rapidly inside cells, allowing monitoring of the subsequent efflux and redistribution of ^64^Cu from all tissues. Significantly enhanced retention of ^64^Cu radioactivity was observed in brain, lungs and blood at 15 h post-injection in symptomatic *Npc1*^−*/*−^ transgenic mice compared to wildtype controls. The enhanced retention of ^64^Cu in brain was confirmed by electronic autoradiography, particularly in the midbrain, thalamus, medulla and pons regions. Positron emission tomography imaging with ^64^Cu in selected chemical forms could be a useful diagnostic and research tool for the management and understanding of NPC1 disease.

## Introduction

Niemann-Pick disease type C (NPC) is an autosomal recessive lysosomal storage disorder resulting from mutations in the *NPC1* (95% of cases) or *NPC2* genes. It is characterised by the accumulation of lysosomal cholesterol and sphingolipids giving rise to liver dysfunction and progressive neurodegeneration, including cerebellar atrophy leading to ataxia. Additionally, NPC1 disease patients have been shown to develop Alzheimer’s disease-like neurofibrillary pathology, which also contributes to the neurodegenerative process (Love et al. 1995; Auer et al. 1995; Vanier 2010; Neufeld et al. 1999; Davies et al. 2000; Liscum 2000). Miglustat is approved as a disease-modifying therapy that slows disease progression (Sévin et al. 2007; Patterson et al. 2007).

In vitro and in vivo studies have demonstrated that abnormal lipid metabolism in NPC1 disease is accompanied by oxidative stress (Reddy et al. 2006; Klein et al. 2011; Smith et al. 2009; Zampieri et al. 2009; Zhang et al. 2008; Porter et al. 2010; Zhang et al. 2014; Klinke et al. 2015). Metal dyshomeostasis (notably copper) in NPC1 disease has been reported and may enhance reactive oxygen species (ROS) production and accelerate neurodegeneration (Vázquez et al. 2012a, b; Sakiyama et al. 2014; Connemann et al. 2012). The NPC1 protein may play a direct role in normal copper metabolism since it has been reported to mediate the incorporation of endosomal copper into ceruloplasmin in cultured hepatocytes (Yanagimoto et al. 2009, 2011). However, copper imbalance in NPC1 disease may merely be a consequence of aberrant lipid storage since copper and cholesterol metabolic pathways have been shown to be interconnected (Hung et al. 2013). Whole-brain copper levels have not been measured in human brain tissue, but were found to be decreased in NPC1 null mice compared to healthy controls (Hung et al. 2014). Copper content in the cerebellum was shown to be reduced or unchanged in NPC1 null mice relative to age-matched controls (Vázquez et al. 2012b, Hung et al. 2014). Copper levels in cerebrospinal fluid were found to be decreased in a very small cohort of NPC1 patients (n = 2) as compared with healthy subjects (Hung et al. 2014). Thus, reports on central nervous system (CNS) copper content in NPC are sparse and there is no clear consensus. Recently, a patient with copper overload (thought initially to be suffering from Wilson’s disease following the discovery of elevated copper in a liver biospy) was correctly rediagnosed with NPC, suggesting that tissue copper levels may be altered in NPC1 patients (Connemann et al. 2012). An improved understanding of copper trafficking and tissue distribution in NPC1 disease is therefore needed and would greatly facilitate (i) the development of new diagnostic techniques, (ii) the elucidation of the mechanism of disease pathogenesis and progression and (iii) the design of more effective therapies, including potential chelation therapy.

The increased availablity of positron emission tomography (PET) and positron-emitting radioisotopes of biologically important metals has made PET an attractive method for tracking trace metal fluxes both in humans and animal models (Bartnicka and Blower 2018). In this study we have explored for the first time the value of PET with the positron-emitting radioisotope ^64^Cu to image short-term copper trafficking in healthy *Npc1*^+*/*+^ versus presymptomatic and symptomatic *Npc1*^−*/*−^ mice (6- and 9-week-old). The aim was to delineate potential abnormalities in copper trafficking, both in the CNS and at whole-body level, that might serve as a biomarker for the disorder. This novel approach enables dynamic, live, non-invasive investigation of Cu fluxes in the same mouse over time and could be readily extended to humans. Intravenous (i.v.) injection of ^64^Cu in the form of an ionic Cu(II) salt gives rise to radiocopper uptake in brain and spinal cord that is insufficient for PET imaging of uptake processes (Andreozzi et al. 2017). Instead, here we study copper retention and efflux of radiocopper delivered directly to cells and tissues, bypassing specific delivery processes by using ^64^Cu-GTSM [glyoxalbis(*N*^*4*^-methyl-3-thiosemicarbazonato) copper(II)], an uncharged, lipophilic Cu(II) complex that displays excellent blood–brain barrier (BBB) penetration and is able to release its Cu payload efficiently and nonselectively into cells (Torres et al. 2016; Fodero-Tavoletti et al. 2010).

The BALB/cNctr-Npc1^m1N^/J (*Npc1*^−*/*−^) null mutant mouse model of NPC1 disease is well-characterised and displays an early-onset, severe form of NPC1 disease, with lifespan limited to 11–12 weeks (Shang et al. 2013; Loftus et al. 1997; Morris et al. 1982; Shio et al. 1982; Bhuvaneswaran et al. 1982). Like NPC1 patients, NPC1 deficient (*Npc1*^−*/*−^) mice develop progressive neurodegeneration characterised by cerebral atrophy, hypomyelination and degeneration of cerebellar Purkinje cells (Voikar et al. 2002). A 6-weeks of age time point was chosen as representative of the presymptomatic/early stage of the disease, while 9-week-old mice represent the symptomatic phase of the disease (Shang et al. 2013).

## Materials and methods

### Animals

All animal experiments were performed in accordance with the Animals (Scientific Procedures) Act, 1986 and ARRIVE guidelines, under a UK Home Office Licence (PPL70/7459). Animals were kept in standard conditions and fed ad libitum with regular animal feed (PicoLab Rodent Diet 20 EXT IRR, LabDiet ^®^, US). BALB/cNctr-*Npc1*^*m1N*^/J mutant mice (The Jackson Laboratory, Charles River, UK) were maintained by heterozygote sibling matings and genotyped to identify *Npc1*^−*/*−^ (null) and wildtype (*Npc1*^+*/*+^, WT) mice as previously described (Loftus et al. 1997). Food intake, natural behaviour and animal body weight were monitored weekly. At the appropriate age, animals (all from the same source; technical replicates) were assigned to groups and transported from the Oxford laboratory to the King’s laboratory for the imaging study and acclimatised for one week prior to imaging. Only female mice were used for the in vivo studies. Data acquisition and analysis were performed without blinding.

### Radiochemistry

Glyoxalbis(*N*^*4*^-methyl-3-thiosemicarbazone) (H_2_-GTSM) was synthesised following a previously reported procedure (Torres et al. 2016). ^64^Cu was produced as previously reported on a CTI RDS 112 biomedical cyclotron at the Clinical PET Centre of St Thomas’ Hospital in the form of ^64^CuCl_2_ in 0.1 N HCl solution (4.8 GBq/μg). Radioactivity was measured using a Capintec CRC-25R dose calibrator. Radio-TLC was performed on a LabLogic Flow-Count scanner (scan speed: 0.25 mm/s), using MERCK 60 F_254_ silica gel TLC plates as the stationary phase and ethyl acetate as the mobile phase.

^64^CuCl_2_ (~ 100 MBq, pH 1, 0.5 mL) was buffered to pH 6 with a 3 M solution of sodium acetate (Sigma-Aldrich). To this solution, 10 µg of H_2_-GTSM in dimethyl sulfoxide (DMSO, 10 μL, 1 mg/mL, Sigma-Aldrich) were added. The reaction mixture was vortexed for 1 min and allowed to stand at room temperature for 5–10 min. The resulting ^64^Cu-GTSM solution was then loaded onto a C_18_ cartridge (Sep-Pak C_18_ Plus Short Cartridge, 360 mg Sorbent, 55–105 µm particle size, Waters Ltd.), previously conditioned with ethanol and water. The sample was washed through with water (5 mL) and ethanol (0.3 mL) and the product collected by elution with a further 1 mL ethanol (~ 90% isolated radiochemical yield, ≥ 95% radiochemical purity). For intravenous administration, ^64^Cu-GTSM was diluted in sterile 0.9% saline solution (w/v) to < 10% of total injected volume (< 200 μL).

### PET/CT imaging

Imaging experiments were performed using a nano-Scan^®^ PET/CT scanner (Mediso Medical Imaging Systems, Budapest, Hungary). ^64^Cu-GTSM-PET was performed in 6- and 9-week-old female *Npc1*^−*/*−^ mice and age-matched WT controls (n = 7 in 9-week-old *Npc1*^−*/*−^ group, n = 6 in 6-week-old *Npc1*^−*/*−^ group, n = 5 in WT groups). All animals were anaesthetised by isoflurane inhalation (3%, Vet Tech Solutions Ltd.) for immobilisation and injected with ^64^Cu-GTSM (~ 15 to 20 MBq, ≤ 200 µL) via a lateral tail vein. Immediately after injection, mice were placed on the scan bed in the prone position and imaged for 30 min by PET/CT (PET: 400-600 keV energy window, 5 ns coincidence window, 0.30 × 0.30 × 0.30 mm^3^ voxel size; CT: 180 projections, 45 KVp, 0.25 × 0.25 × 0.21 mm^3^ voxel size). Anaesthesia was maintained with 1.5-2% isoflurane throughout the duration of the scan. Respiration rate and bed temperature were monitored during image acquisition. After imaging, mice were allowed to recover and housed overnight. At 15 h post-injection of ^64^Cu-GTSM, animals were re-scanned as described above.

### PET image analysis

All PET/CT datasets were reconstructed using 3D iterative algorithms (8 iterations, 0.25 × 0.25 × 0.25 mm^3^ voxel size) (Magdics et al. 2011). Intercrystal scatter correction was applied. All PET images were decay-corrected to injection time prior to quantification. All reconstructed datasets were analysed using VivoQuant 1.21 software (Invicro, LLC, Boston, USA). PET and CT images were co-registered and fixed-volume 3D ROIs (~ 15 mm^3^) were drawn in organs of interest to measure ^64^Cu concentration. The sum of activity (MBq) in the organ ROI was divided by the sum of activity (MBq) in the whole-body ROI (excluding tail) at the time of injection and the resulting percentage [% injected dose (ID)] was normalised by the volume of the organ ROI to express ^64^Cu uptake as %ID/mL. Global and regional concentrations of ^64^Cu in the brain were measured using a mouse brain atlas NM/CT module (Invicro, LLC).

### Ex vivo biodistribution

After the 15 h imaging time point, all mice were culled and dissected. Organs of interest were explanted, weighed and gamma-counted (LKB Wallac 1282) to measure ^64^Cu concentration in each organ as %ID/g. The sum of whole-body counts (excluding tail) and excreted activity (urine and faeces) was considered as the total ID.

### Ex vivo brain autoradiography

After dissection and gamma counting, brain tissue was cryopreserved in 30% sucrose solution for ~ 6 h, flash-frozen in isopentane (Sigma-Aldrich) and stored at − 80 °C overnight. Sagittal 10 µm brain sections were then cut using a Bright 5040 cryotome and thaw-mounted onto Superfrost PLUS glass microscope slides (Menzel-Glaser, Thermo Scientific), which were then exposed to a storage phosphor screen (PerkinElmer, Super Resolution, 12.5 × 25.2 cm) for 15 h. Finally, the screen was scanned using a Typhoon 8600 scanner (Molecular Dynamics) and the resulting autoradiographs were analysed with OptiQuant 5.0 (PerkinElmer) and ImageJ (NIH).

### Statistical analysis

In exploratory studies such as this where expected difference size and variance is unknown, the number of animals per group in our imaging experiments is typically estimated using a two-tailed *T* test with a confidence interval of 95% and a power of 80%. For a signal-to-noise ratio of 2 ± 1 versus 7.5 ± 4.5 after injection, to detect a nominal difference of 5.5 for a nominal standard deviation of 4.5, 8 animals per group need to be studied. Our group size was determined by approaching this ideal as closely as resources allowed and with a pre-set minimum of 3 per group, while avoiding breeding unnecessarily large numbers. Sample size differences are the result of sorting after genotyping; data from all available animals were included in the analysis. All data are reported as mean ± standard deviation (SD). Statistical analysis was performed using GraphPad Prism 5 (GraphPad Software Inc.). Significance of differences between groups was estimated with two-tailed Student’s t tests and Mann–Whitney U tests. Two-way repeated measures ANOVA with post hoc analyses using the Bonferroni multiple comparison correction was used to determine statistically significant differences between *Npc1*^−*/*−^ mice and WT controls at different imaging time-points. No data were defined as outliers.

## Results

### PET/CT imaging

In order to investigate short-term (< 15 h) copper trafficking in NPC1 disease, *Npc1*^−*/*−^ mice at 6 and 9 weeks of age and age-matched WT controls were intravenously injected with ^64^Cu-GTSM and live imaged from 0 to 30 min and from 15 to 15.5 h post-injection.

**Fig. 1:**
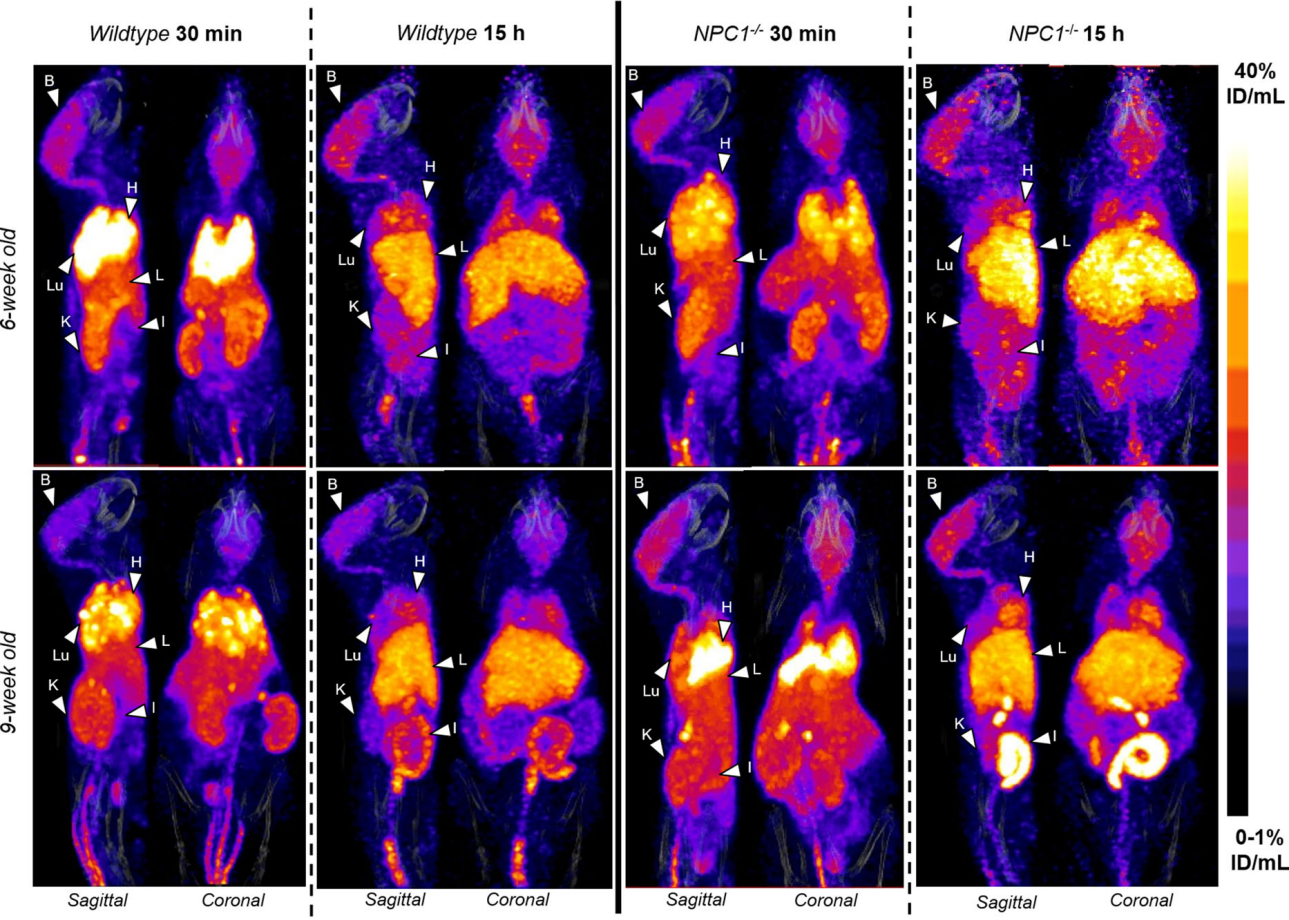
Representative sagittal and coronal views of PET/CT images (maximum intensity projection, MIP) of 6-week-old (top) and 9-week-old (bottom) female WT (left) and *Npc*^−*/*−^ (right) mice at 30 min (left) and 15 h (right) post-injection of ^64^Cu-GTSM. PET images in all four groups were visually similar, showing high accumulation of ^64^Cu radioactivity in the heart (H), lungs (Lu), liver (L), kidneys (K), adrenals, intestines (I) and brain (B) at 30 min after injection of the tracer in all mice. The spinal cord was also clearly visible. While ^64^Cu accumulation decreased in the heart, lungs, adrenals and kidneys between 30 min and 15 h, the concentration of radiocopper in the liver and intestines increased. Brain uptake of ^64^Cu remained relatively constant over time. All MIPs were scaled to 0–1%ID/mL (min) and 40%ID/mL (max)

Figure 1, as an example, depicts the in vivo biodistribution of ^64^Cu in 6- and 9-week-old *Npc1*^−*/*−^ mice and WT controls at 30 min and 15 h after injection At 30 min post-injection of the tracer the images were qualitatively similar for all four groups (6 week-old *Npc1*^−*/*−^, 6 week-old WT, 9 week-old *Npc1*^−*/*−^, 9 week-old WT), showing that ^64^Cu accumulated predominantly in the lungs, heart and to a lesser extent in the liver, intestines, kidneys, and brain. In addition, the images showed marked uptake in adrenal glands and spinal cord, organs that were not harvested ex vivo. At 15 h, the images for all four groups were also qualitatively similar but the activity in the lungs, kidneys, heart and adrenal glands was visibly diminished compared to 30 min, while uptake in brain, spinal cord and liver was not visibly different.

**Fig. 2:**
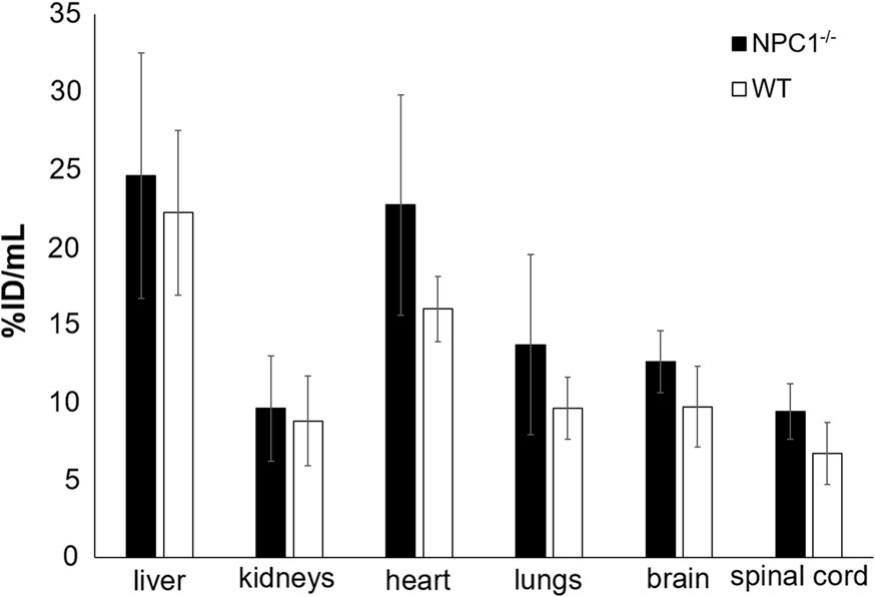
Uptake (%ID/mL) of ^64^Cu in organs distinguishable on PET scans of 9-week-old *Npc1*^−*/*−^ (red bars, n = 7) and WT (blue bars, n = 5) mice 15 h post injection of ^64^Cu-GTSM, determined by quantitative image analysis. Error bars represent ± one standard deviation. The same trends are evident as in Fig. 7 but the standard deviations are larger because of the intrinsic limitations of PET region of interest quantification and volume determination

These visual impressions were confirmed by quantitative image analysis, from which uptake values were obtained in organs of interest (Fig. 2). Although the volume of smaller organs is overestimated by the method used to define ROIs because of the partial volume effect (and consequently the %ID/mL values may be underestimated), these values were similar to those obtained from ex vivo biodistribution in 9-week-old mice at 15 h (vide infra). The %ID/mL values for lung derived from PET were much less than the %ID/g values derived from ex vivo organ counting because the density of lung is much less than 1.0 g/mL, unlike other tissues. The standard deviations of the PET-derived uptake values were, however, higher than those of the values derived from ex vivo organ counting. Consequently, while the uptake in hearts, lungs and brains of 9-week-old *Npc1*^−*/*−^ mice was higher than that in WT mice (by 42, 43 and 29% respectively) whether measured by PET or ex vivo organ counting, the differences measured by PET did not reach statistical significance. Uptake in spinal cord, as measured by PET, was also higher in 9-week-old *Npc1*^−*/*−^ mice than in their WT counterparts (by 39%).

**Fig. 3:**
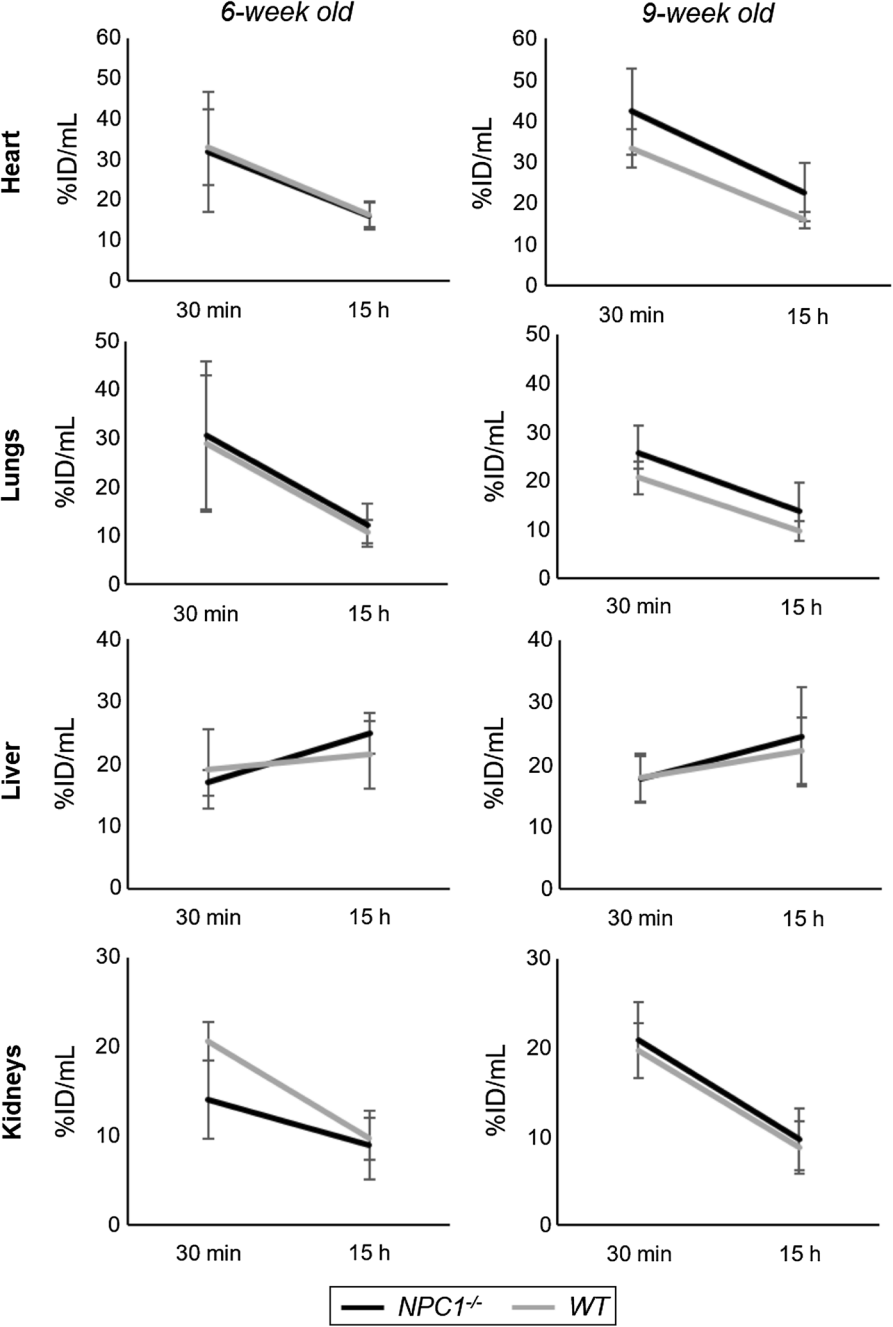
Dynamics of radioactivity uptake and efflux in heart, lungs, kidney and liver, showing uptake of ^64^Cu at 30 min and 15 h post injection of ^64^Cu-GTSM in 6-week-old (left) and 9-week-old (right) *Npc1*^−*/*−^ (black, n = 7) and WT (grey, n = 5) mice. Error bars represent ± one standard deviation

PET imaging allows investigation of the dynamics of the tracer distribution during the 15 h post injection. Figure 3 shows significant net efflux of radioactivity between 30 min and 15 h from heart (a percentage change in radioactivity of − 40 ± 26% in *Npc1*^−*/*−^ mice, − 47 ± 19% in WT mice at 6 weeks; − 47 ± 11% in *Npc1*^−*/*−^ mice, − 51 ± 10% in WT mice at 9 weeks), lungs (− 55 ± 21% in *Npc1*^−*/*−^ mice, − 59% ± 13% in WT mice at 6 weeks; − 42 ± 33% in *Npc1*^−*/*−^ mice, − 53 ± 9% in WT mice at 9 weeks) and kidneys (− 48 ± 10% in *Npc1*^−*/*−^ mice, − 53 ± 12% in WT mice at 6 weeks; − 54 ± 11% in *Npc1*^−*/*−^ mice, − 56 ± 8% in WT mice at 9 weeks). The liver, by contrast, showed a net influx of radioactivity between 30 min and 15 h (+ 48 ± 21% in *Npc1*^−*/*−^ mice, + 20% ± 38% in WT mice at 6 weeks; + 43 ± 45% in *Npc1*^−*/*−^ mice, + 32 ± 52% in WT mice at 9 weeks). However, this time-dependent change in liver activity was subject to large variation in all four groups, ranging from + 77 to − 50% in individual animals. The % efflux/influx in these organs of *Npc1*^−*/*−^ mice was not significantly different to that in WT mice.

**Fig. 4:**
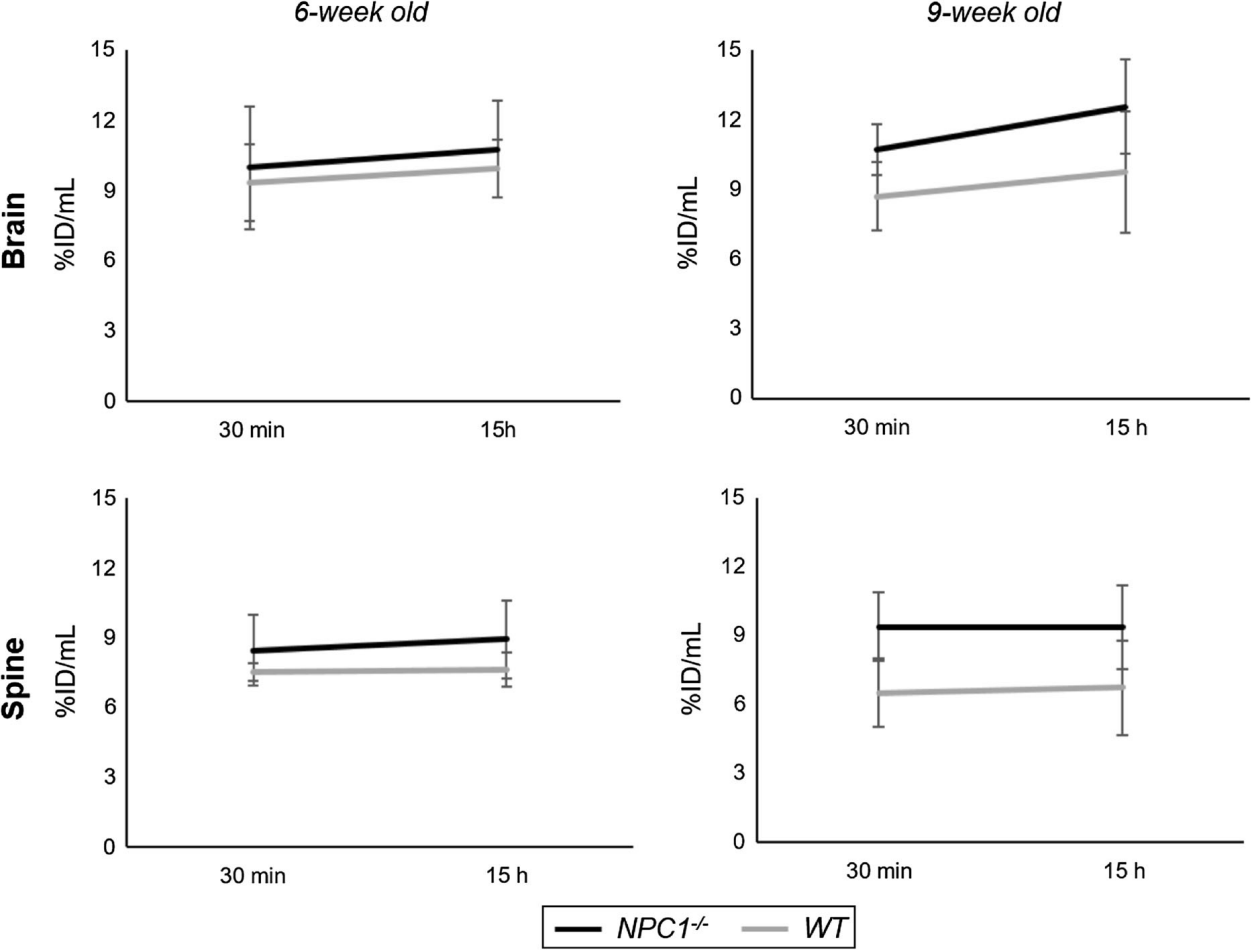
Dynamics of radioactivity uptake and efflux in brain and spinal cord, showing uptake of ^64^Cu at 30 min and 15 h post injection of ^64^Cu-GTSM in 6-week-old (left) and 9-week-old (right) *Npc1*^−*/*−^ (black, n = 7) and WT (grey, n = 5) mice. Error bars represent ± one standard deviation

In marked contrast to heart, lungs, liver and kidneys, the changes in radioactivity levels in the CNS between 30 min and 15 h (Fig. 4) were minor and not significant, showing that activity accumulated by 30 min was retained; indeed, there was a small net increase of activity in the brain during the period (10 ± 10% in *Npc1*^−*/*−^ mice, 8 ± 9% in WT mice at 6 weeks; 17 ± 17% in *Npc1*^−*/*−^ mice, 11 ± 12% in WT mice at 9 weeks). Similarly, in spinal cord there was no significant net efflux; indeed a marginal (non-statistically significant) net influx between 30 min and 15 h (+ 6 ± 10% in *Npc1*^−*/*−^ mice, + 1 ± 6% in WT mice at 6 weeks; 0 ± 7% in *Npc1*^−*/*−^ mice, + 2 ± 9% in WT mice at 9 weeks).

**Fig. 5:**
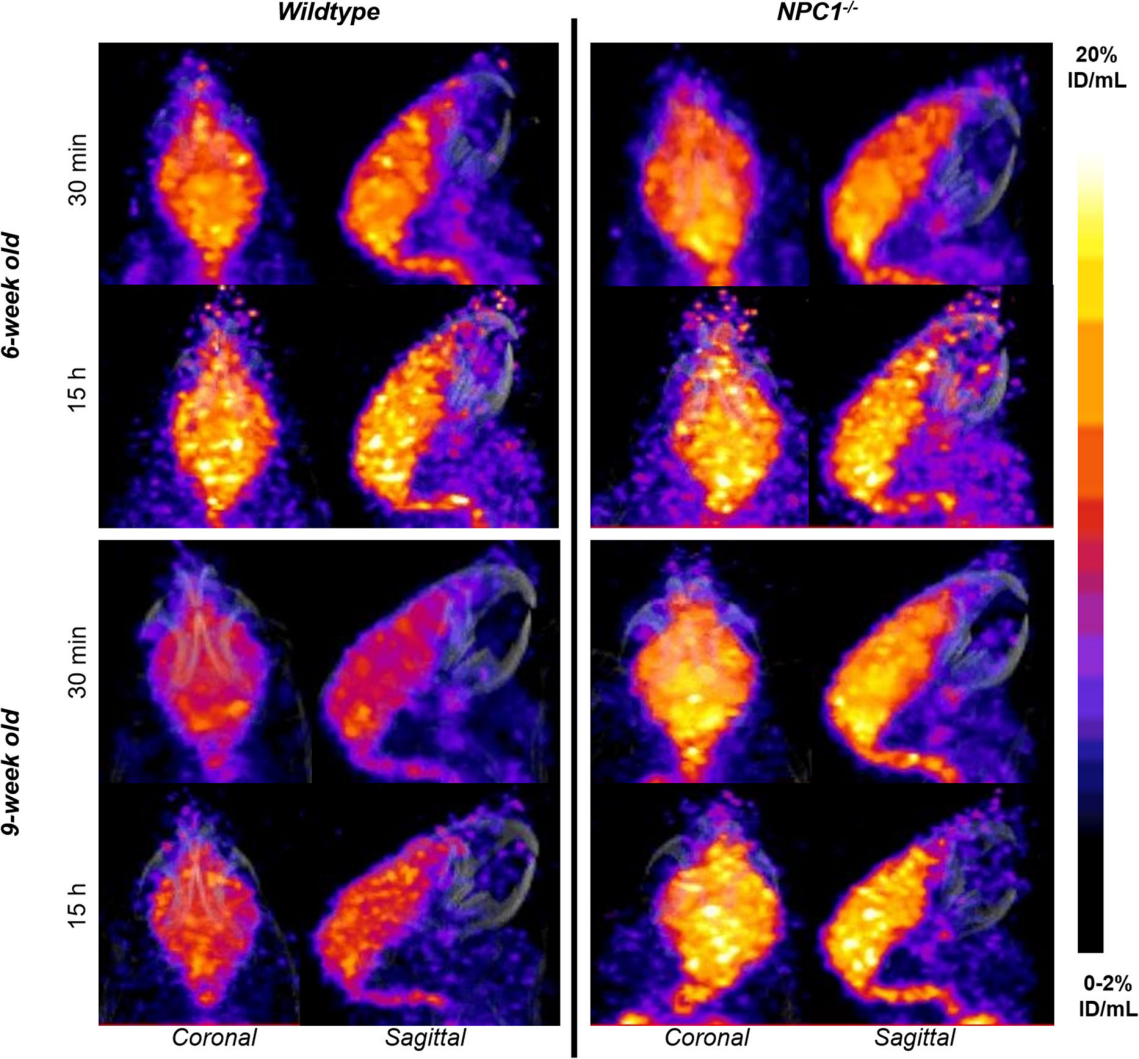
Representative coronal and sagittal PET/CT MIP images illustrating ^64^Cu distribution in the brains of 6-week-old (upper panel) and 9-week-old (lower panel) WT controls left and age-matched Npc1^−/−^ mice (right) at 30 min (top) and 15 h (bottom) post-injection of ^64^Cu-GTSM. Nine-week-old *Npc*1^−/−^ mice exhibited higher brain ^64^Cu accumulation than their WT littermates at all imaging time-points. All MIPs were scaled to 0–2%ID/mL (min) and 20%ID/mL (max)

Despite the limitations of resolution of PET (of the order of 0.5–0.7 mm), imaging and quantitative analysis of the distribution of radioactivity within the brain was possible. Because of its low positron energy, ^64^Cu provides superior resolution images compared to many other radionuclides used for PET. The anatomical definition of brain regions was based on mapping of the images onto a three-dimensional digital mouse brain atlas. Figure 5 shows examplar images of brains from all four groups. Inhomogenous distribution of radioactivity within the brain was evident in all four groups both at 30 min and 15 h, and it is likely that the inhomogeneity is visually underestimated because of limited resolution of the PET scanner.

**Fig. 6:**
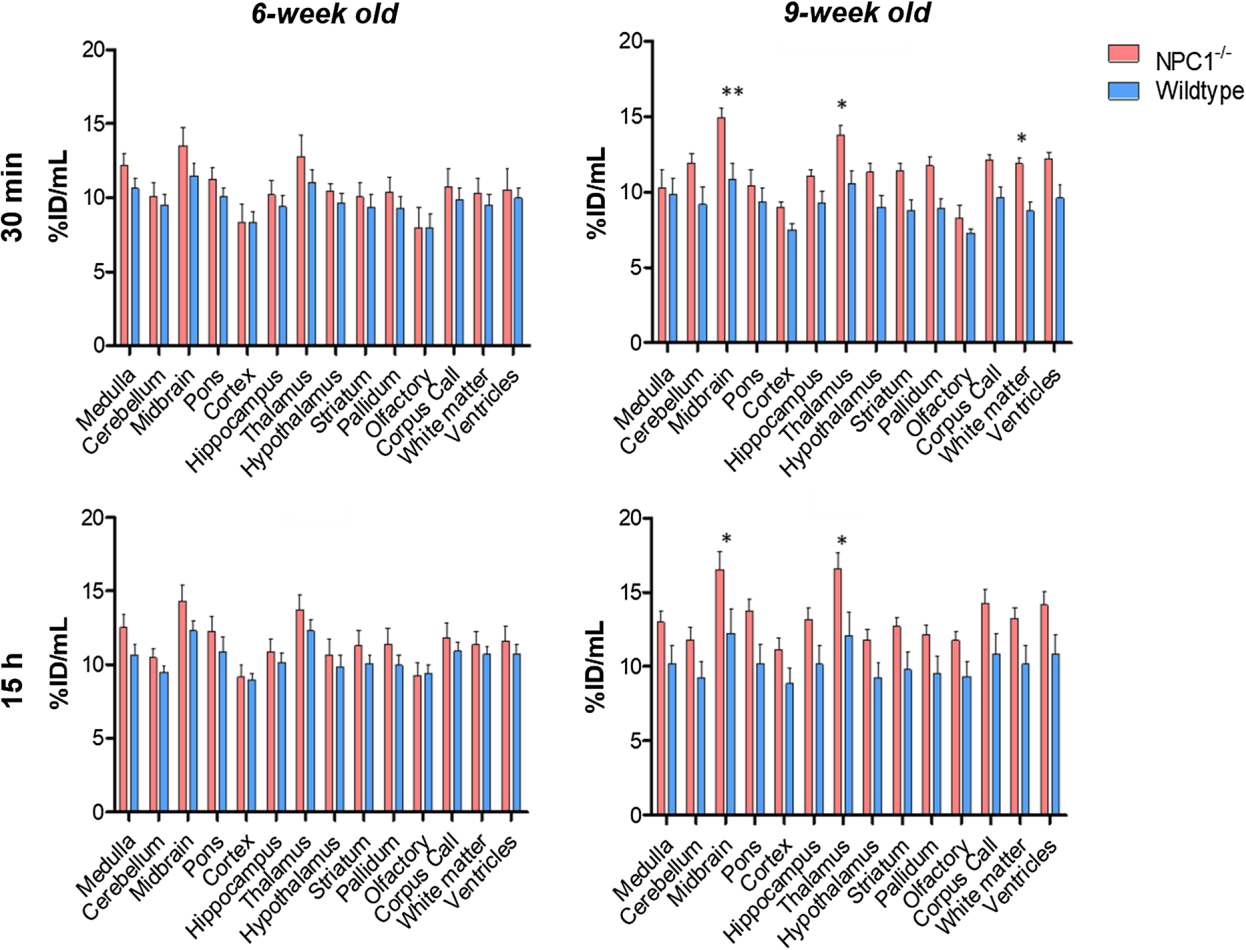
Top: Regional brain distribution of ^64^Cu in 6-week-old (left panels) and 9-week-old (right panels) *Npc1*^−*/*−^ mice and age-matched controls at 30 min (top panels) and 15 h (bottom panels) post-injection of ^64^Cu-GTSM. Data are mean [n = 6-7 *Npc1*^−*/*−^, n = 5 WT)] ± SD. Statistically significant differences from healthy controls were evident in the 9-week-old groups and are indicated by * (*p *< 0.05) and ** (p < 0.01)

The results of quantitative regional analysis mapped onto the mouse brain atlas are shown in Fig. 6. The increased global brain uptake of radioactivity in *Npc1*^−*/*−^ mice compared to WT mice was apparent in all anatomical regions examined and was statistically significant in the 9-week-old group. In all four groups, the regions of highest activity at both 30 min and 15 h were thalamus and midbrain and the regions of lowest activity were the cortex, olfactory bulb and cerebellum. In all groups, there were no significant changes in distribution among brain regions between 30 min and 15 h.

*Post*-*hoc* evaluation revealed significant differences in ^64^Cu concentration in specific brain regions between the *Npc1*^−*/*−^ and WT 9-week-old groups at both imaging time-points (Fig. 6). At 30 min after injection, *Npc1*^−*/*−^ mice exhibited significantly greater ^64^Cu levels than their WT littermates in the midbrain (*p *< 0.01), thalamus (*p *< 0.05) and white matter (*p *< 0.05); these significant differences persisted at 15 h in midbrain (*p *< 0.05) and thalamus (*p *< 0.05). In 6-week-old mice, the same trends were observed but regional differences did not reach statistical significance.

To show that these differences in ^64^Cu concentration in the CNS between *Npc1*^−*/*−^ and WT 9-week-old mice were not affected by subtle differences in body weight between groups, ^64^Cu uptake was also expressed as standardised uptake values (SUV,^1^ which implicitly corrects for body weight). SUV measurements revealed significantly higher ^64^Cu concentration in the spine (p < 0.01; 30 min and 15 h) and brain (p < 0.01; 15 h) of 9-week old *Npc1*^−*/*−^ mice, as well as enhanced ^64^Cu retention in midbrain and thalamus (p < 0.05; 30 min) and thalamus and hypothalamus (p < 0.05; 15 h) when compared to controls.

### Ex vivo biodistribution

**Fig. 7:**
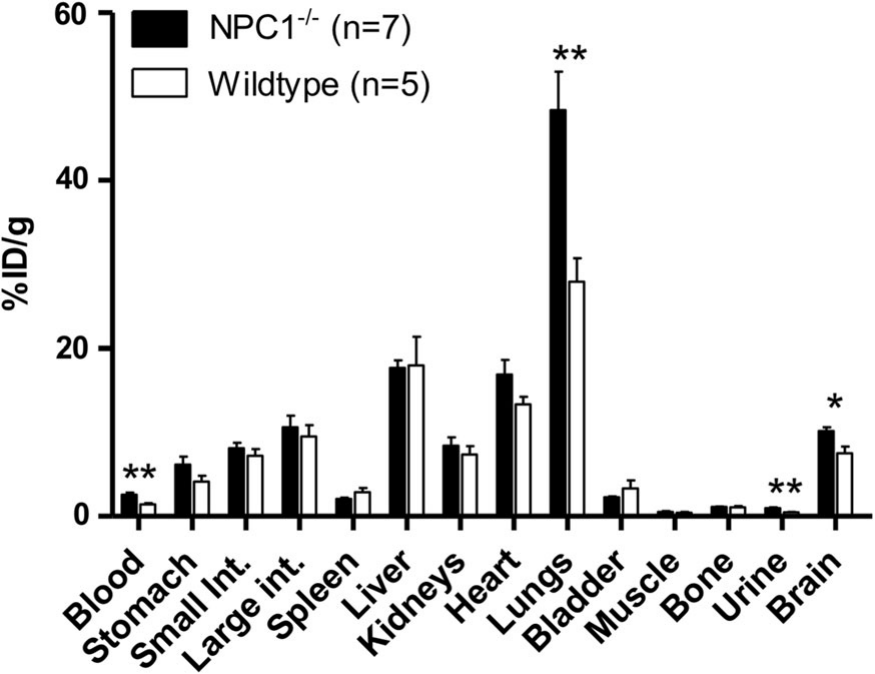
Radioactivity accumulation/retention in harvested organs of 9-week-old *Npc1*^−*/*−^ (black bars, n = 7) and WT (white bars, n = 5) mice, 15 h after i.v. injection of ^64^Cu-GTSM, determined by ex vivo gamma counting. Error bars represent one standard deviation; **p < 0.01; *p < 0.05

In both *Npc1*^−*/*−^ and WT 9-week old mice, ex vivo biodistribution of radioactivity at 15 h post-injection of ^64^Cu-GTSM was measured by counting and weighing harvested organs *post mortem* after the imaging. A high retention (> 20%ID/g) was found in the lungs, liver, heart, intestines and brain (Fig. 7), similar to that observed previously in WT mice (Andreozzi et al. 2017; Torres et al. 2016). Significantly enhanced retention was observed in blood (by 78%, *p* = 0.0043), lungs (by 73%, *p* = 0.005) and brain (by 35%, *p* = 0.03) of *Npc1*^−*/*−^ mice compared to WT mice (Fig. 7). Activity in urine was very low at this late time point but was significantly higher in *Npc1*^−*/*−^ than in WT mice (*p* = 0.0015). Differences in other tissues were not statistically significant.

### Ex vivo brain autoradiography

**Fig. 8:**
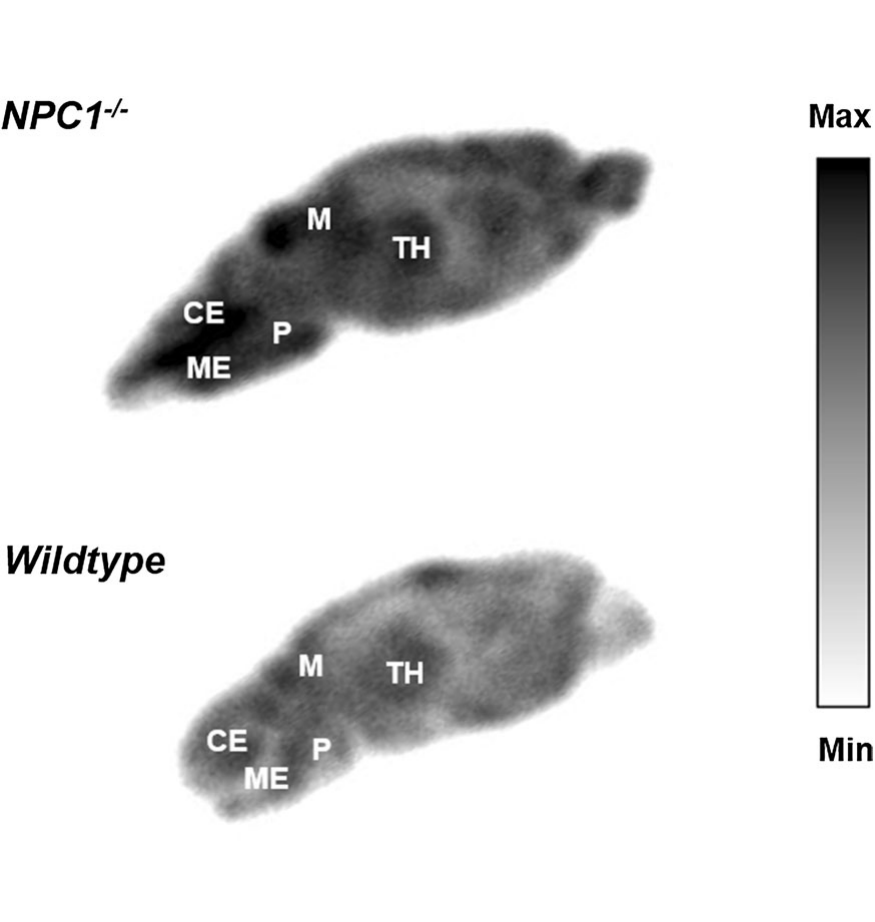
*Ex vivo* autoradiographs from representative sagittal brain sections of 9-week-old *Npc1*^−*/*−^ (top) and WT control (bottom) mice sacrificed at 15 h post-injection of ^64^Cu-GTSM. The main radioactive hotspots in the *Npc1*^−*/*−^ brain autoradiographs were located in the thalamus, midbrain, cerebellum, medulla and pons areas. *TH* thalamus, *M* midbrain, *CE* cerebellum, *ME* medulla, *P* pons

In order to generate high-resolution maps of the distribution of ^64^Cu within brain tissue, sagittal brain sections of *Npc1*^−*/*−^ mice and WT controls sacrificed at 15 h post-injection of ^64^Cu-GTSM were imaged by phosphor imaging autoradiography. Images in Fig. 8 show the regional distribution of ^64^Cu in representative sagittal brain slices of *Npc1*^−*/*−^ mice and WT controls at 15 h after injection of the tracer as measured by digital autoradiography. These ex vivo autoradiographs reflect the heterogeneous distribution of radioactivity within the brain, and the globally higher accumulation of ^64^Cu in the brains of 9-week-old *Npc1*^−*/*−^ mice compared to age-matched WT controls, that were detected by PET imaging. The most prominent radioactive hotspots in the *Npc1*^−*/*−^ brain autoradiographs were found in the midbrain, thalamus, medulla and pons regions.

## Discussion

In this study, we have investigated in vivo copper trafficking in a mouse model of NPC1 disease at presymptomatic (6 weeks) and symptomatic (9 weeks) stages of the disorder by PET imaging using ^64^Cu-GTSM in order to identify any measurable changes in copper efflux and distribution associated with the pathology. We have focused particularly on the delineation of copper trafficking within the CNS since transition metal imbalance has been linked to enhanced oxidative stress in NPC1 disease and is therefore proposed to contribute to the neurodegenerative process characteristic of this disorder (Vázquez et al. 2011, 2012a; Porter et al. 2010). For this reason, we used ^64^Cu-GTSM as a vehicle to deliver intravenously-administered radioactive copper into the CNS and release it within cells, allowing subsequent monitoring of its efflux and trafficking. This mode of administration bypasses the innate absorption phase of endogenous copper metabolism and facilitates rapid deposition of radiocopper within the CNS. However, the fate of orally administered radiocopper should also be evaluated by PET in future studies in order to detect any potential abnormalities in the gastrointestinal absorption and subsequent transport of copper that might be associated with NPC1 disease.

As expected from a previous study (Torres et al. 2016), ^64^Cu-GTSM rapidly delivered radiocopper to all major organs after injection, including the heart, lungs, liver, intestines, kidneys, brain and spinal cord. The decrease of radioactivity in the heart, lungs and kidneys between 30 min and 15 h, and the accompanying accumulation in the liver and intestines, reflects excretion of radiocopper particularly via the biliary system. Significant increases in uptake (whether expressed as  %ID/g or SUV) of ^64^Cu in several tissues were found in *Npc1*^−*/*−^ compared to WT animals. However, despite previous studies reporting increased copper content in the liver of *Npc1* deficient mice compared to control animals (Vázquez et al. 2011; Argüello et al. 2014; Hung et al. 2014), no significant differences in hepatic ^64^Cu concentration were found between *Npc1*^−*/*−^ and WT groups at either 6 or 9 weeks of age. This discrepancy might be explained on the basis of differences between long-term (total copper content measurements) and short-term (acute trafficking studies described here) copper biodistribution; the previously reported increased copper levels in the liver of *Npc1*^−*/*−^ mice are the result of prolonged accumulation of dietary copper in this organ, and might therefore not be measurable by acute PET measurements.

As previously observed both in healthy mice and models of Alzheimer’s disease (Torres et al. 2016; Fodero-Tavoletti et al. 2010), both brain and spinal cord showed surprisingly high radiocopper sequestration. The negligible clearance of ^64^Cu radioactivity from brain and spinal cord between 30 min and 15 h compared to other tissues, in all groups, might be caused by a particularly high metabolic demand for copper of the CNS and/or less active or absent copper efflux mechanisms compared to other tissues.

Differences in whole-brain and spinal cord ^64^Cu concentration between presymptomatic (6-week-old) *Npc1*^−*/*−^ mice and age-matched controls were observed but did not reach statistical significance. However, the difference persisted and increased with age such that at 9 weeks, *Npc1*^−*/*−^ mice exhibited significantly higher ^64^Cu concentration (whether expressed as %ID/g or SUV) than WT controls at both 30 min and 15 h post-injection of ^64^Cu-GTSM. Interestingly, these differences in brain and spinal cord ^64^Cu concentration between the *Npc1*^−*/*−^ and WT groups were associated with a significant decrease in brain ^64^Cu concentration with age in these organs in WT mice.

Reduced total copper content observed in the cerebellum of NPC1-deficient mice (Hung et al. 2014) contrasts with the increased acute ^64^Cu uptake/retention in our experiments, highlighting the importance of measuring both long-term chronic total copper accumulation and its short-term trafficking. Copper levels in the remaining brain regions have not previously been reported.

The finding of significantly higher ^64^Cu concentration in the blood of *Npc1*^−*/*−^ mice 15 h post injection than their WT littermates is in agreement with previous research revealing increased copper content in blood plasma in presymptomatic and symptomatic *Npc1*^−*/*−^ mice compared to healthy controls (Vázquez et al. 2012b; Hung et al. 2014). This elevated blood ^64^Cu concentration (by 78%) in *Npc1*^−*/*−^ mice would not fully account for the enhanced accumulation of ^64^Cu (by 35%) in their brains and spinal cords, because blood radioactivity is much lower than that of brain and spinal cord, and blood content in these tissue is not high. Elevation of plasma copper in NPC1-deficient mice has been associated with an increase in plasma ceruloplasmin levels, which might indicate a higher rate of synthesis/secretion of this protein in NPC1 disease (Vázquez et al. 2012b; Hung et al. 2014). NPC1 belongs to a family of proteins termed RND permeases that have been implicated in bacterial systems to play a role in metal ion transport (Kim et al. 2011). NPC1 may therefore directly play a role in copper export from the lysosome (Yanagimoto et al. 2009, 2011) or indirectly affect other transport systems involved in copper transport/homeostasis (Argüello et al. 2014) and merits further investigation.

Additional studies are needed to determine whether plasma ^64^Cu is inserted into ceruloplasmin or bound to other plasma proteins (e.g. albumin, transcuprein), and whether increased blood ^64^Cu concentration in *Npc1*^−*/*−^ mice can be detected at earlier time-points after injection of the tracer (< 15 h). The relationship between blood copper concentration and disease severity and progression also needs to be evaluated in order to determine the potential of this parameter as an early diagnostic marker of NPC1 disease.

## Conclusions

This study has demonstrated the potential of ^64^Cu-GTSM-PET as a tool to identify alterations in copper metabolism associated with NPC disease processes, particularly within the CNS. ^64^Cu-GTSM-PET revealed significantly increased ^64^Cu concentration in the brains and spinal cords of symptomatic *Npc1*^−*/*−^ mice compared to healthy controls at both 30 min and 15 h post-injection of the tracer. Symptomatic *Npc1*^−*/*−^ mice exhibited significantly higher ^64^Cu concentration in blood than their WT littermates at 15 h after injection. Despite the statistical power of this study being insufficient to confirm some of the subtler changes in copper trafficking, the origin of these metabolic abnormalities along with their relationship with disease severity and progression merit further investigation. Due to the inability of NPC1-deficient mouse models to mimic the phenotypic variability of human NPC1 disease, PET imaging of copper trafficking should additionally be performed in human subjects in order to delineate any potential abnormalities that could be used to assess the status of the disease and potentially monitor response to treatment.

## Footnotes

1 %ID/g for the specified tissue divided by the %ID for the whole body, excluding tail.

## References

[CR1] Andreozzi EM, Torres JB, Sunassee K, Dunn J, Walker-Samuel S, Szanda I, Blower PJ (2017). Studies of copper trafficking in a mouse model of Alzheimer’s disease by positron emission tomography: comparison of ^64^Cu acetate and ^64^CuGTSM. Metallomics.

[CR2] Argüello G, Martinez P, Peña J, Chen O, Platt F, Zanlungo S, González M (2014). Hepatic metabolic response to restricted copper intake in a Niemann-Pick C murine model. Metallomics.

[CR3] Auer I, Schmidt M, Lee V-Y, Curry B, Suzuki K, Shin R-W, Pentchev P, Carstea E, Trojanowski J (1995). Paired helical filament tau (PHFtau) in Niemann-Pick type C disease is similar to PHFtau in Alzheimer’s disease. Acta Neuropathol.

[CR4] Bartnicka JJ, Blower PJ (2018). Insights into trace metal metabolism in health and disease from PET: PET metallomics. J Nucl Med.

[CR5] Bhuvaneswaran C, Morris MD, Shio H, Fowler S (1982). Lysosome lipid storage disorder in NCTR-BALB/c mice. III. Isolation and analysis of storage inclusions from liver. Am J Pathol.

[CR6] Connemann BJ, Gahr M, Schmid M, Runz H, Freudenmann RW (2012). Low ceruloplasmin in a patient with Niemann-Pick Type C disease. J Clin Neurosci.

[CR7] Davies JP, Chen FW, Ioannou YA (2000). Transmembrane molecular pump activity of Niemann-Pick C1 protein. Science.

[CR8] Fodero-Tavoletti MT, Villemagne VL, Paterson BM, White AR, Li QX, Camakaris J, O’Keefe G, Cappai R, Barnham KJ, Donnelly PS (2010). Bis(thiosemicarbazonato) Cu-64 complexes for positron emission tomography imaging of Alzheimer’s disease. J Alzheimers Dis.

[CR9] Hung YH, Bush AI, La Fontaine S (2013). Links between copper and cholesterol in Alzheimer’s disease. Front Physiol.

[CR10] Hung YH, Faux NG, Killilea DW, Yanjanin N, Firnkes S, Volitakis I, Ganio G, Walterfang M, Hastings C, Porter FD, Ory DS, Bush AI (2014). Altered transition metal homeostasis in Niemann-Pick disease, type C1. Metallomics.

[CR11] Kim EH, Nies DH, McEvoy MM, Rensing C (2011). Switch or funnel: How RND-type transport systems control periplasmic metal homeostasis. J Bacteriol.

[CR12] Klein A, Maldonado C, Vargas LM, Gonzalez M, Robledo F, Perez de Arce K, Muñoz FJ, Hetz C, Alvarez AR, Zanlungo S (2011). Oxidative stress activates the c-Abl/p73 proapoptotic pathway in Niemann-Pick type C neurons. Neurobiol Dis.

[CR13] Klinke G, Rohrbach M, Giugliani R, Burda P, Baumgartner MR, Tran C, Gautschi M, Mathis D, Hersberger M (2015). LC-MS/MS based assay and reference intervals in children and adolescents for oxysterols elevated in Niemann-Pick diseases. Clin Biochem.

[CR14] Liscum L (2000). Niemann-Pick type C mutations cause lipid traffic jam. Traffic.

[CR15] Loftus SK, Morris JA, Carstea ED, Gu JZ, Cummings C, Brown A, Ellison J, Ohno K, Rosenfeld MA, Tagle DA, Pentchev PG, Pavan WJ (1997). Murine model of Niemann-Pick C disease: mutation in a cholesterol homeostasis gene. Science.

[CR16] Love S, Bridges LR, Case CP (1995). Neurofibrillary tangles in Niemann—Pick disease type C. Brain.

[CR17] Morris MD, Bhuvaneswaran C, Shio H, Fowler S (1982). Lysosome lipid storage disorder in NCTR-BALB/c mice. I. Description of the disease and genetics. Am J Pathol.

[CR19] Magdics M, Szirmay-Kalos L, Tóth B, Légrády D, Cserkaszky Á, Balkay L, Domonkos B, Völgyes D, Patay, G, Major P, Lantos J and Bükki T (2011) Performance evaluation of scatter modeling of the GPU-based Tera-Tomo; 3D PET reconstruction. In: Nucl Sci Symp Med Imaging Conf (NSS/MIC), pp 4086–4088. IEEE. 10.1109/NSSMIC.2011.6153777

[CR18] Neufeld EB, Wastney M, Patel S, Suresh S, Cooney AM, Dwyer NK, Roff CF, Ohno K, Morris JA, Carstea ED, Incardona JP, Strauss JF, Vanier MT, Patterson MC, Brady RO, Pentchev PG, Blanchette-Mackie EJ (1999). The Niemann-Pick C1 protein resides in a vesicular compartment linked to retrograde transport of multiple lysosomal cargo. J Biol Chem.

[CR20] Patterson MC, Vecchio D, Prady H, Abel L, Wraith JE (2007). Miglustat for treatment of Niemann-Pick C disease: a randomised controlled study. Lancet Neurol.

[CR21] Porter FD, Scherrer DE, Lanier MH, Langmade SJ, Molugu V, Gale SE, Olzeski D, Sidhu R, Dietzen DJ, Fu R, Wassif CA, Yanjanin NM, Marso SP, House J, Vite C, Schaffer JE, Ory DS (2010). Cholesterol oxidation products are sensitive and specific blood-based biomarkers for Niemann-Pick C1 disease. Sci Transl Med.

[CR22] Reddy JV, Ganley IG, Pfeffer SR (2006). Clues to neuro-degeneration in Niemann-Pick type C disease from global gene expression profiling. PLoS ONE.

[CR23] Sakiyama Y, Narita A, Osawa S, Nanba E, Ohno K, Otsuka M (2014). Abnormal copper metabolism in Niemann-Pick disease type C mimicking Wilson’s disease. Neurol Clin Neurosci.

[CR24] Sévin M, Lesca G, Baumann N, Millat G, Lyon-Caen O, Vanier MT, Sedel F (2007). The adult form of Niemann-Pick disease type C. Brain.

[CR25] Shang XY, Lin XJ, Manorek G, Howell SB (2013). Claudin-3 and claudin-4 regulate sensitivity to cisplatin by controlling expression of the copper and cisplatin influx transporter CTR1. Mol Pharmacol.

[CR26] Shio H, Fowler S, Bhuvaneswaran C, Morris M (1982). Lysosome lipid storage disorder in NCTR-BALB/c mice. II. Morphologic and cytochemical studies. Am J Pathol.

[CR27] Smith D, Wallom KL, Williams IM, Jeyakumar M, Platt FM (2009). Beneficial effects of anti-inflammatory therapy in a mouse model of Niemann-Pick disease type C1. Neurobiol Dis.

[CR28] Torres JB, Andreozzi EM, Dunn JT, Siddique M, Szanda I, Howlett DR, Sunassee K, Blower PJ (2016). PET imaging of copper trafficking in a mouse model of Alzheimer disease. J Nucl Med.

[CR29] Vanier MT (2010). Niemann-Pick disease type C. Orphanet J Rare Dis.

[CR30] Vázquez MC, del Pozo T, Robledo FA, Carrasco G, Pavez L, Olivares F, González M, Zanlungo S (2011). Alteration of gene expression profile in Niemann-Pick type C mice correlates with tissue damage and oxidative stress. PLoS ONE.

[CR31] Vázquez MC, Balboa E, Alvarez AR, Zanlungo S (2012). Oxidative stress: a pathogenic mechanism for Niemann-Pick type C disease. Oxid Med Cell Longev..

[CR32] Vázquez MC, Martínez P, Alvarez AR, González M, Zanlungo S (2012). Increased copper levels in in vitro and in vivo models of Niemann-Pick C disease. Biometals.

[CR33] Voikar V, Rauvala H, Ikonen E (2002). Cognitive deficit and development of motor impairment in a mouse model of Niemann-Pick type C disease. Behav Brain Res.

[CR34] Yanagimoto C, Harada M, Kumemura H, Koga H, Kawaguchi T, Terada K, Hanada S, Taniguchi E, Koizumi Y, Koyota S, Ninomiya H, Ueno T, Sugiyama T, Sata M (2009). Niemann-Pick C1 protein transports copper to the secretory compartment from late endosomes where ATP7B resides. Exp Cell Res.

[CR35] Yanagimoto C, Harada M, Kumemura H, Abe M, Koga H, Sakata M, Kawaguchi T, Terada K, Hanada S, Taniguchi E, Ninomiya H, Ueno T, Sugiyama T, Sata M (2011). Copper incorporation into ceruloplasmin is regulated by Niemann-Pick C1 protein. Hepatol Res.

[CR36] Zampieri S, Mellon SH, Butters TD, Nevyjel M, Covey DF, Bembi B, Dardis A (2009). Oxidative stress in NPC1 deficient cells: protective effect of allopregnanolone. J Cell Mol Med.

[CR37] Zhang JR, Coleman T, Langmade SJ, Scherrer DE, Lane L, Lanier MH, Feng C, Sands MS, Schaffer JE, Semenkovich CF, Ory DS (2008). Niemann-Pick C1 protects against atherosclerosis in mice via regulation of macrophage intracellular cholesterol trafficking. J Clin Invest.

[CR38] Zhang H, Wang Y, Lin N, Yang R, Qiu W, Han L, Ye J, Gu X (2014). Diagnosis of Niemann-Pick disease type C with 7-ketocholesterol screening followed by NPC1/NPC2 gene mutation confirmation in Chinese patients. Orphanet J Rare Dis.

